# Supplementary material for: Substrate structure and computation guided engineering of a lipase for omega-3 fatty acid selectivity
Source: PLoS One. 2020 Apr 9;15(4):e0231177. doi: 10.1371/journal.pone.0231177 (PMC7145112; doi:10.1371/journal.pone.0231177)
Supplement: S1 Fig — Ramachandran plot of modelled GTL (a) and DM-GTL (b) in lid open conformation. Triangle represents glycine and square represents proline. Other amino acids are represented by circle. Red colour indicates most favoured region, deep yellow indicates additional allowed region and light yellow indicates generously allowed region. (PDF) [file pone.0231177.s001.pdf]

Supplementary materials

Substrate structure and computation guided engineering of a Lipase for Omega-3 fatty acid selectivity

Tushar Ranjan Moharana and Nalam Madhusudhana Rao*
CSIR-Centre for Cellular and Molecular Biology
Uppal Road
Hyderabad India 500007

a **b**

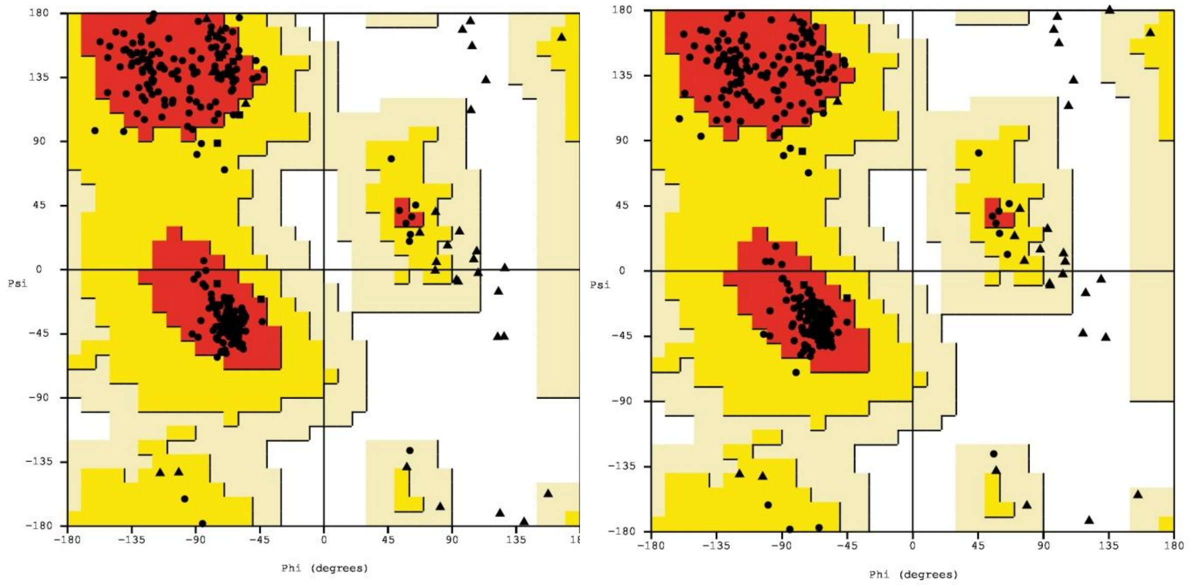

Figure S1: Ramachandran plot of modelled GTL (a) and DM-GTL (b) in lid open conformation.

Triangle represents glycine and square represents proline. Other amino acids are represented by circle. Red colour indicates most favoured region, deep yellow indicates additional allowed region and light yellow indicates generously allowed region.
