# Supplementary material for: Substrate structure and computation guided engineering of a lipase for omega-3 fatty acid selectivity
Source: PLoS One. 2020 Apr 9;15(4):e0231177. doi: 10.1371/journal.pone.0231177 (PMC7145112; doi:10.1371/journal.pone.0231177)
Supplement: S2 Fig — Amino acid positions chosen for SSM were shown in white, while amino acids interacting with substrate, but not included for SSM, are shown in magenta and active serine (S113) is shown in red. Triglyceride molecule shown in green represents substrate as shown in Fig 1A and the molecule shown in yellow represents substrate as shown in Fig 1B. (PDF) [file pone.0231177.s002.pdf]

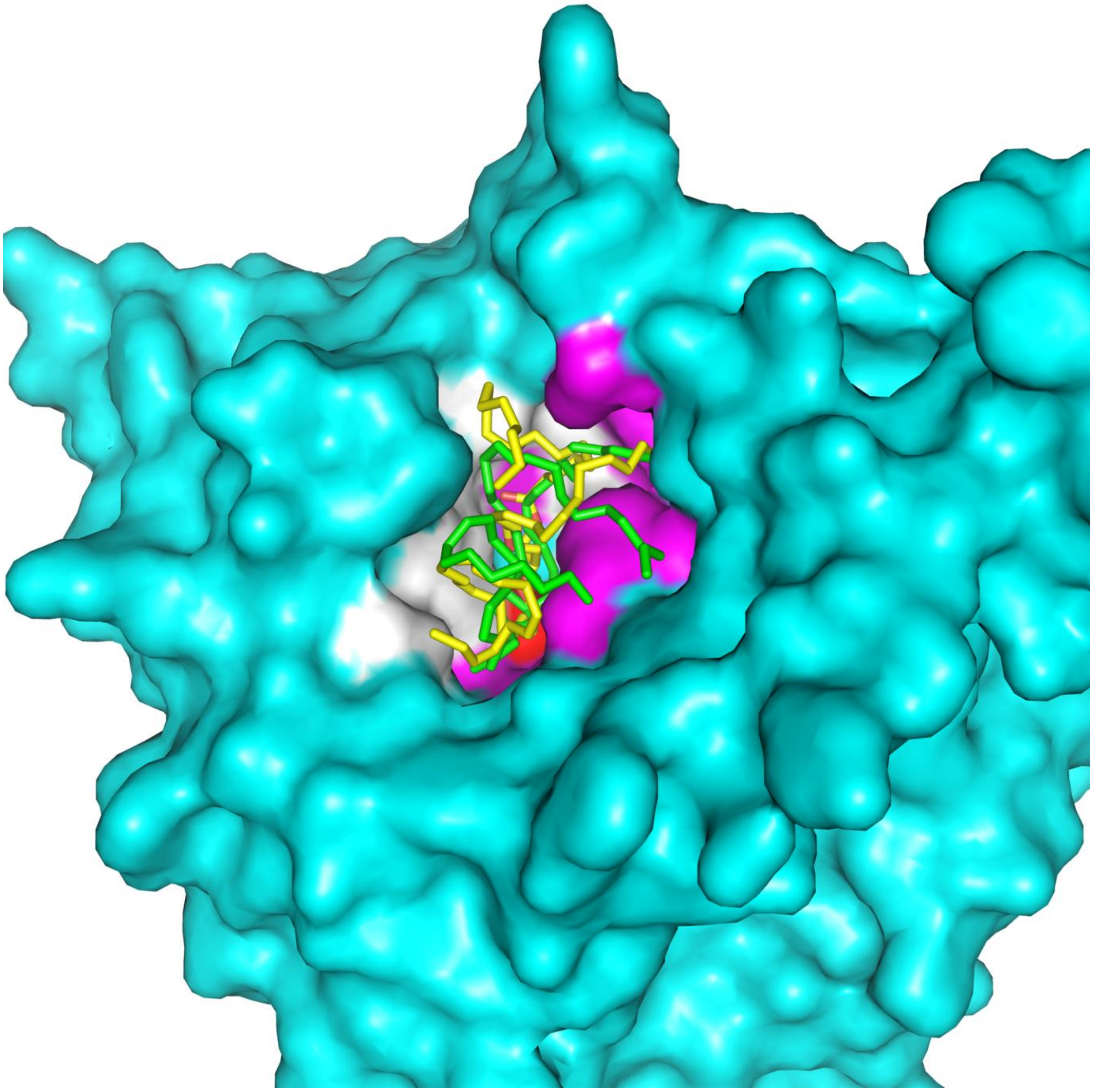

Figure S2: Covalent docking of triglyceride with GTL.
Amino acid positions chosen for SSM were shown in white, while amino acids interacting with substrate, but not included for SSM, are shown in magenta and active serine (S113) is shown in red. Triglyceride molecule shown in green represents substrate as shown in Fig.1a and the molecule shown in yellow represents substrate as shown in Fig.1b.
