# Supplementary material for: Substrate structure and computation guided engineering of a lipase for omega-3 fatty acid selectivity
Source: PLoS One. 2020 Apr 9;15(4):e0231177. doi: 10.1371/journal.pone.0231177 (PMC7145112; doi:10.1371/journal.pone.0231177)
Supplement: S3 Fig — EPA (Light grey) and DHA (Dark). (PDF) [file pone.0231177.s003.pdf]

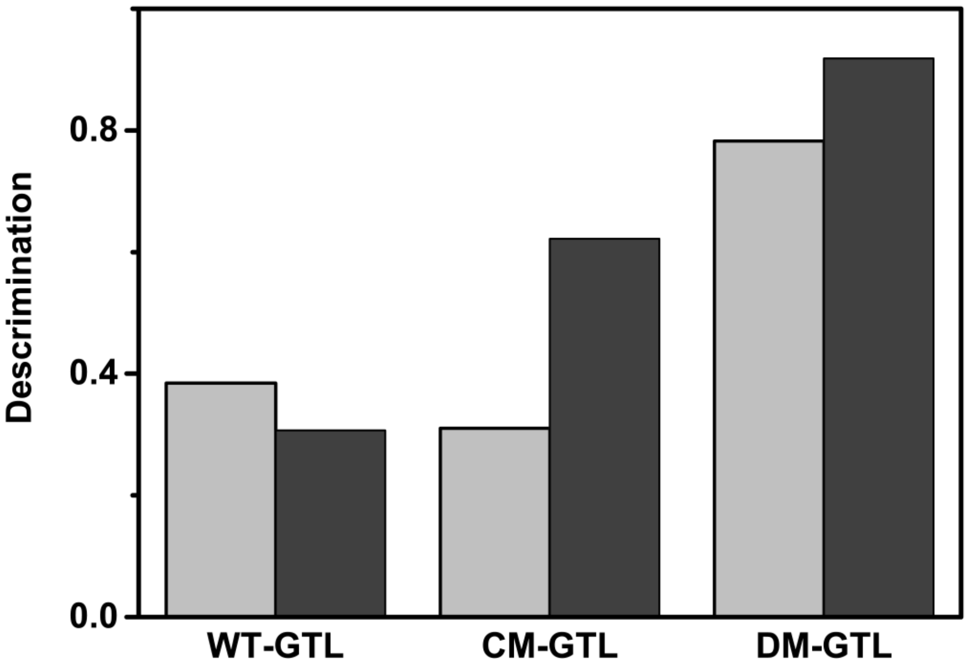

Figure S3: Fatty acid discrimination of GTL, CM-GTL and DM-GTL at 20 percent hydrolysis.
EPA (Light grey) and DHA (Dark).
