# Supplementary material for: Substrate structure and computation guided engineering of a lipase for omega-3 fatty acid selectivity
Source: PLoS One. 2020 Apr 9;15(4):e0231177. doi: 10.1371/journal.pone.0231177 (PMC7145112; doi:10.1371/journal.pone.0231177)
Supplement: S4 Fig — (PDF) [file pone.0231177.s004.pdf]

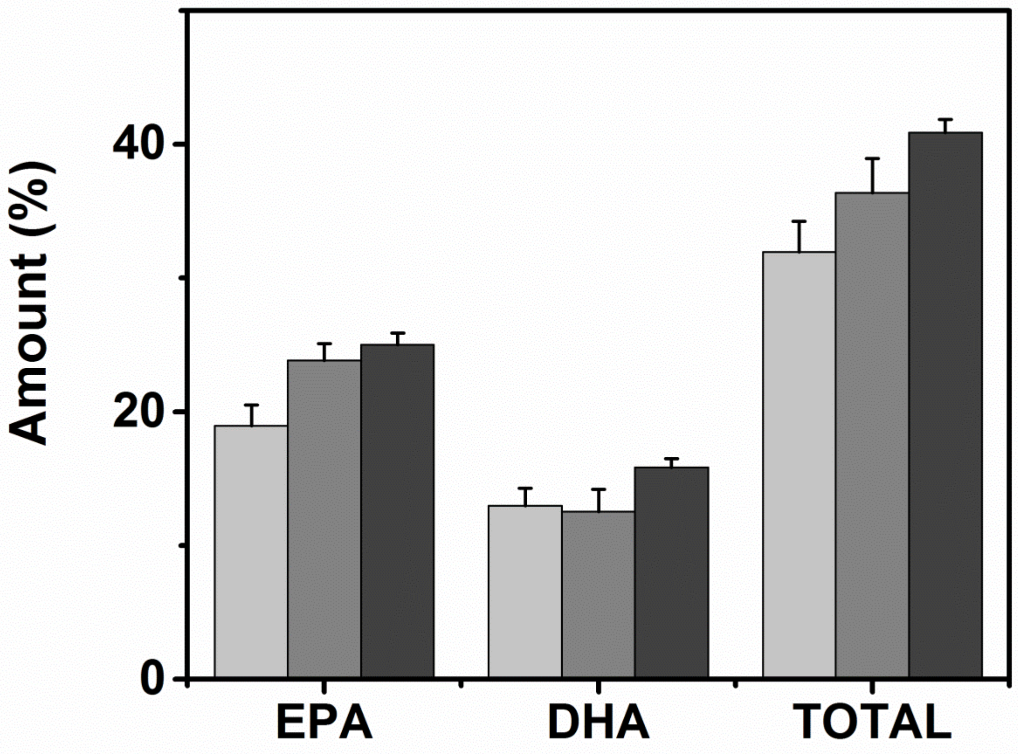


**Figure S4**: **Concentration of EPA and DHA in the glyceride fraction of unhydrolyzed anchovy oil (Light grey bars) and after 20 percent hydrolysis of anchovy oil by GTL (Dark grey bars) and DM-GTL (Black bars).**
