# Supplementary material for: Substrate structure and computation guided engineering of a lipase for omega-3 fatty acid selectivity
Source: PLoS One. 2020 Apr 9;15(4):e0231177. doi: 10.1371/journal.pone.0231177 (PMC7145112; doi:10.1371/journal.pone.0231177)
Supplement: S1 Table — Primers used in SSM (top) and for SDM (bottom). Mutations are shown in bold. (PDF) [file pone.0231177.s005.pdf]

Amino acid position	Forward primer	Reverse primer
170	TGACGGGACGACG**NNK**GTCAACATGGTTG	CAACCATGTTGAC**MNN**CGTCGTCCCGTCA
171	GGACGACGCTT**NNK**AACATGGTTGATTTC	GAAATCAACCATGTT**MNN**AAGCGTCGTCC
244	ATACCGCCCGCTACGAT**NNK**TCCGTTCCC	GGGAACGGA**MNN**ATCGTAGCGGGCGGTAT
319	GAACGACGGC**NNK**GTCAATACCATTTCGA	TCGAAATGGTATTGAC**MNN**GCCGTCGTTC
358	GTCGAC**NNK**TTGGAAATCATCGGCGTTGACCC	GGGTCAACGCCGATGATTTCCAA**MNN**GTCGAC
359	AATGTCGACCAT**NNK**GAAATCATCGGCGT	ACGCCGATGATTTC**MNN**ATGGTCGACATT

Amino acid position	Forward primer	Reverse primer
170 and 171	GACGGGACGACG**GGTTGG**AACATGGTTG	CAACCATGTT**CCAACC**CGTCGTCCCGTC
359	GTCGACCATT**GTG**AAATCATCGGCG	CGCCGATGATTT**CAC**AATGGTCGAC
171	GGACGACGCTT**CTC**AACAT	ATGTT**GAG**AAGCGTCGTCC
183	GCTTTTTTGAC**TTC**ATGAAAGCG	CGCTTTCAT**GAA**GTCAAAAAAGC

Table S1: Primers used in SSM (Top) and for SDM (Bottom).
Mutations are shown in bold.
