# Supplementary material for: Substrate structure and computation guided engineering of a lipase for omega-3 fatty acid selectivity
Source: PLoS One. 2020 Apr 9;15(4):e0231177. doi: 10.1371/journal.pone.0231177 (PMC7145112; doi:10.1371/journal.pone.0231177)
Supplement: S2 Table — Mutants showing improved difference in activity (>2) are sequenced and the mutations were identified. (PDF) [file pone.0231177.s006.pdf]

Plate A anchovy oil score

1	2	3	4	5	6	7	8	9	10	11	12
1	1	4	4	0	2	3	3	3	1	1	4
1	1	3	2	2	3	3	0	1	1	1	1
1	1	1	2	2	2	2	1	2	2	2	2
1	2	1	2	1	1	1	3	2	2	1	1
1	2	1	1	1	4	3	3	1	4	1	1
2	1	3	3	3	2	2	3	3	1	1	1
							2	1	3	3	3

Plate A coconut oil score

1	2	3	4	5	6	7	8	9	10	11	12
1	0	3	3	0	3	4	4	4	3	3	4
1	4	2	3	3	3	3	0	3	2	2	3
1	3	2	2	4	4	3	2	2	2	2	4
2	3	1	4	2	3	3	4	4	3	1	1
1	3	1	2	2	4	4	4	1	4	3	1
2	2	3	3	4	3	2	4	4	2	2	1
							4	3	4	4	4

Plate A difference in score

1	2	3	4	5	6	7	8	9	10	11	12
0	-1	-1	-1	0	1	1	1	1	**2**	**2**	0
0	**3**	-1	1	1	0	0	0	**2**	1	1	**2**
0	**2**	1	0	**2**	**2**	1	1	0	0	0	**2**
1	1	0	**2**	1	**2**	**2**	1	**2**	1	0	0
0	1	0	1	1	0	1	1	0	0	**2**	0
0	1	0	0	1	1	0	1	1	1	1	0
0	0	0	0	0	0	0	**2**	**2**	1	1	1

Plate C anchovy oil score								Plate B anchovy oil score													
5	6	7	8	9	10	11	12		1	2	3	4	5	6	7	8	9	10	11	12	
0	4	3	3		2	3	4	A	0	1	1	2	2	1	2	2	2	1	2	2	A
0	2	2	2	2	2	0	1	B	2	1	1	2	3	2	1	1	1	2	2	2	B
2	0	1	2	2	1	1	4	C	2	1	1	2	2	1	2	2	2	1	2	2	C
0	2		2	2	2		2	D	2	2	2	1	2	2	2	2	1	1	1	0	D
0	2	2	2	2	2	2	2	E	2	1	1	1	1	3	1	2	2	2	2	2	E
					2	2	1	F	0	2	2	1	2	2	2	2	2	2	2	2	F
								G								1	2	1	1	0	G
								H													H
Plate C coconut oil score								Plate B coconut oil score													
5	6	7	8	9	10	11	12		1	2	3	4	5	6	7	8	9	10	11	12	
0	2	2	2	2	2	3	4	A	0	1	1	2	2	1	2	2	2	1	2	2	A
0	2	2	1	2	2	2	2	B	2	1	1	2	3	2	2	2	0	2	2	2	B
2	2	1	2	2	1	1	4	C	2	1	1	3	2	2	2	2	2	3	2	2	C
0	2	1	2	2	2	1	2	D	2	2	2	2	3	2	2	2	3	1	1	2	D
0	2	4	2	2	2	2	1	E	2	2	3	2	2	1	2	2	2	2	2	2	E
					2	2	1	F	1	2	2	2	2	2	2	2	2	2	2	2	F
								G								1	2	3	1	0	G
								H													H
Plate C difference in score								Plate B difference in score													
5	6	7	8	9	10	11	12		1	2	3	4	5	6	7	8	9	10	11	12	
0	-2	-1	-1	**2**	0	0	0	A	0	0	0	0	0	0	0	0	0	0	0	0	A
0	0	0	-1	0	0	**2**	1	B	0	0	0	0	0	0	1	1	-1	0	0	0	B
**2**	**2**	0	0	0	0	0	0	C	0	0	0	1	0	1	0	0	0	**2**	0	0	C
0	0	1	0	0	0	1	0	D	0	0	0	1	**2**	0	0	0	**2**	0	0	**2**	D
0	0	**2**	0	0	0	0	-1	E	0	1	**2**	1	1	-2	1	0	0	0	0	0	E
0	0	0	0	0	0	0	0	F	1	0	0	1	0	0	0	0	0	0	0	0	F
								G	0	0	0	0	0	0	0	0	0	**2**	0	0	G
								H													H

Plate D anchovy oil score	1	2	3	4	5	6	7	8	9	10	11	12
A	2	2	2	2	2	2	2	2	1	2	2	3
B								2	2	1	1	0
C												
D												
E												
F												
G												
H												

	1	2	3	4
A	1	1	0	1
B	1	1	2	2
C	1	2	1	2
D	2	2	2	2
E	1	2	1	2
F				
G				
H				

Plate D coconut oil score	1	2	3	4	5	6	7	8	9	10	11	12
A	2	2	2	2	2	2	2	2	3	2	2	2
B								2	2	0	2	1
C												
D												
E												
F												
G												
H												

	1	2	3	4
A	1	2	0	2
B	1	1	2	2
C	1	2	1	2
D	2	2	2	2
E	1	2	1	1
F				
G				
H				

Plate D difference in score	1	2	3	4	5	6	7	8	9	10	11	12
A	0	0	0	0	0	0	0	0	**2**	0	0	-1
B	0	0	0	0	0	0	0	0	0	-1	1	1
C												
D												
E												
F												
G												
H												

	1	2	3	4
A	0	1	0	1
B	0	0	0	0
C	0	0	0	0
D	0	0	0	0
E	0	0	0	-1
F	0	0	0	0
G				
H				

Table S2: Activity of GTL mutants on anchovy and coconut oil.
Mutants showing improved difference in activity (>2) are sequenced and the mutations were identified.
